# The prevalence of primary headache disorders in Nepal: a nationwide population-based study

**DOI:** 10.1186/s10194-015-0580-y

**Published:** 2015-11-10

**Authors:** Kedar Manandhar, Ajay Risal, Timothy J Steiner, Are Holen, Mattias Linde

**Affiliations:** Department of Neuroscience, Norwegian University of Science and Technology, St Olavs University Hospital, Trondheim, Norway; Dhulikhel Hospital, Kathmandu University Hospital, Kavre, Dhulikhel, Nepal; Division of Brain Sciences, Imperial College London, London, UK; Pain Unit, St Olavs University Hospital, Trondheim, Norway; Norwegian Advisory Unit on Headaches, St Olavs University Hospital, Trondheim, Norway

**Keywords:** Migraine, Tension-type headache, Medication-overuse headache, Public health, population-based study, prevalence, Nepal, South-East Asia Region, Global campaign against headache

## Abstract

**Background:**

Headache disorders are among the most prevalent and burdensome global public-health problems. Within countries, health policy depends upon knowledge of health within the local populations, but the South-East Asia Region (SEAR), among WHO’s six world regions, is the only one for which no national headache prevalence data are available.

**Methods:**

In a cross-sectional population-based study, adults representative of the Nepali-speaking population aged 18–65 years and living in Nepal were randomly recruited using stratified multistage cluster sampling. They were visited unannounced at home by trained interviewers who used a culturally-adapted Nepali translation of the structured Headache-Attributed Restriction, Disability, Social Handicap and Impaired Participation (HARDSHIP) questionnaire.

**Results:**

There were 2,100 participants (1,239 females [59.0 %], 861 males [41.0 %]; mean age 36.4 ± 12.8 years) with 9 refusals (participation rate 99.6 %). Over half (1,100; 52.4 %) were resident above 1,000 m and almost one quarter (470; 22.4 %) lived at or above 2,000 m. The 1-year prevalence of any headache was 85.4 ± 1.5 % (gender- and age-adjusted 84.9 %), of migraine 34.7 ± 2.0 % (34.1 %), of tension-type headache (TTH) 41.1 ± 2.1 % (41.5 %), of headache on ≥15 days/month 7.7 ± 1.1 % (7.4 %) and of probable medication-overuse headache (pMOH) 2.2 ± 0.63 % (2.1 %).

There was a strong association between migraine and living at altitude ≥1,000 m (AOR = 1.6 [95 % CI: 1.3-2.0]; *p* < 0.001). There was a less strong association between TTH and urban dwelling (AOR = 1.3 [95 % CI: 1.1-1.6]; *p* = 0.003), and a possibly artefactual negative association between TTH and living above 1,000 m (AOR = 0.7 [95 % CI: 0.6-0.8]; *p* < 0.001).

**Conclusion:**

Headache disorders are very common in Nepal. Migraine is unusually so, and strongly associated with living at altitude, which in very large part accounts for the high national prevalence: the age- and gender- standardised prevalence in the low-lying Terai is 27.9 %. Headache occurring on ≥15 days/month is also common. This new evidence will inform national health policy and provide a basis for health-care needs assessment. However, research is needed to explain the association between migraine and altitude, since it may be relevant to health-care interventions.

## Background

Headache disorders are among the most prevalent, burdensome and costly diseases in the world [1]. The primary headache disorders, mostly migraine and tension-type headache (TTH), are of importance to global public health because they lead to widespread ill health and impaired quality of life [2], and important to global economies because they also cause much loss of productivity [3]. Improper treatment of migraine or TTH can lead to medication-overuse headache (MOH), an aggravated disorder which, by definition, occurs on ≥15 days/month and is a major additional contributor to global disability. The Global Burden of Disease Study 2013 (GBD2013) found migraine to be the sixth highest cause of disability worldwide and MOH the 18^th^ in terms of years of life lost to disability (YLDs) [4]. Collectively, headache disorders rank third [5].

Headache prevalence is poorly described in many large and populous regions of the world. Nowhere is this more obvious than in the South-East Asia Region (SEAR), the only one of the World Health Organization’s six world regions for which no nationwide data have yet been gathered about the prevalence of headache disorders or their impact on society [6].

Within SEAR, Nepal is one of the poorest countries [7]. Its population is approximately 30 million [8], of whom about one quarter live below the international poverty line and among the others the distribution of wealth is rather unequal [7]. Furthermore, Nepal is a country of wide diversity. More than 70 ethnic groups maintain different cultures and spoken languages [9]. Topographically the country is divided into three physiographic divisions – Terai, Hill and Mountain [10] – rising from some 60 m above sea level to the Himalayas, including Mount Everest, the highest peak on Earth at 8,848 m [7], to attest Nepal’s extraordinary geographical variation.

Although headache has been found to be one of the most common complaints among patients in primary health-care centres in Nepal [11, 12], no epidemiological studies have established the prevalence of headache disorders in this country. Epidemiological data are required to inform policy and decide the efficient allocation of resources in a country such as Nepal which has a very limited health-care budget. With this purpose, the aim of our study was two-fold: 1) to estimate the prevalence of headache disorders of public-health importance: migraine, TTH and MOH; and 2) to explore demographic and environmental factors associated with these headache disorders in Nepal. The study was conducted as a project within the Global Campaign against Headache, which is led by *Lifting The Burden* (LTB), a UK-registered non-governmental organisation in official relationship with the World Health Organization.

## Methods

### Ethics

The Nepal Health Research Council, the Institutional Review Committee of Kathmandu University School of Medical Sciences, Dhulikhel Hospital (IRC-KUSMS) and the Regional Committee for Health and Research Ethics in central Norway all approved the study protocol.

All participants were informed about the nature and purpose of the study. Written consent was obtained by signature or fingerprint in accordance with requirements of IRC-KUSMS.

### Study design

This was a cross-sectional, population-based survey using structured interviews administered by trained health workers making unannounced door-to-door visits to households in May, 2013. We randomly selected one eligible adult (aged 18–65 years, Nepali-speaking and living in Nepal) from each household. To ensure adequate representation from the country as a whole, we used multistage stratified cluster sampling, including all three physiographic divisions and, within each, all five development regions (Far-Western, Midwestern, Western, Central and Eastern). The details of the sampling and data collection procedure, including the steps taken to ensure a very high participation rate, have been published elsewhere [10]. The sample size (N = 2,100) was estimated assuming a headache-type prevalence of ≥10 % and absolute margin of error of 1.3 % with 95 % confidence interval (CI).

### Instruments

We used the Headache-Attributed Restriction, Disability, Social Handicap and Impaired Participation (HARDSHIP) questionnaire developed by LTB for similar studies [13]. The English version was translated into Nepalese according to LTB’s translation protocol for lay documents [14] and adapted to fit the characteristics of the Nepalese culture [15].

The full questionnaire has been published previously [10]. It consisted of five parts. For all participants there were (i) personal and demographic enquiry and (ii) a headache screening question (“*Have you had a headache in the last year?*”). Those who answered “*no*” were classified as headache-free. Those who answered “*yes*” were asked whether their headaches were of one or more types and, if more than one, to focus only on the most bothersome type. Only those who answered positively to the screening question were also asked (iii) diagnostic and (iv) burden and health-care questions relating to their headache. To the standard HARDSHIP questions we added others relating to use of herbal therapies. Finally, there were (v) questions on certain comorbidities asked of all participants [10].

We used culturally-validated Nepali-translations of the Hospital Anxiety and Depression Scale (HADS) [16] and the Eysenck Personality Questionnaire Neuroticism Short Form Revised version (EPQRS-N) [17] to assess psychiatric comorbidity. We measured height, weight and waist-circumference, and calculated body-mass index (BMI). We measured blood pressure (BP) using a digital device (3BM1-3® by Microlife).

We recorded the altitude of each household using a portable altimeter (SAL 7030® by Sunoh).

### Headache diagnosis

Diagnoses were not made during the interviews but later by an algorithm [13]. Participants reporting headache on ≥15 days/month were first separated as a distinct group because they cannot be fully diagnosed by questionnaire. Those who were also overusing acute medication were considered to have probable MOH (pMOH); the remainder were diagnosed as “other headache on ≥15 days/month”. Medication overuse was diagnosed in those who: a) reported the use on ≥15 days/month of either a single class of analgesics, or one type of herbal medicine, as acute headache treatment; or b) reported using on ≥10 days/month (i) a combination of analgesics of different classes, or (ii) more than one type of herbal medicine, or (iii) a combination of analgesics and herbal medicines. Triptans and ergots were not used.

To all others, reporting headache on ≤14 days/month, the algorithm applied modified criteria of the International Classification of Headache Disorders (ICHD-3 beta) [18] in the following order: definite migraine, definite TTH, probable migraine and probable TTH. We found that two additional adaptations were necessary. Firstly, photophobia was reported in association with more than three-quarters (75.8 %) of all headaches and therefore offered little discriminative value diagnostically. Accordingly, we ignored it when diagnosing headache types. Secondly, according to ICHD-3 beta [18], attacks lasting <4 h when untreated in adults may be compatible with a diagnosis of probable migraine when other criteria are met. Many of our participants could report attack durations only after taking acute medication, and some were very short. We decided to disallow a diagnosis of probable migraine (in favour of probable TTH) whenever headache duration was <1 h. We took the view that so short a duration, even with acute treatment, was very unlikely to be migraine, given that the adult participant was asked to describe a “usual” attack [19].

Cases of definite and probable migraine were combined as, likewise, were cases of definite and probable TTH in the estimations of prevalence and in association analyses. Remaining cases were considered unclassifiable.

### Statistics

We estimated crude 1-year prevalence with 95 % CI for all headache, migraine and TTH, and point prevalence for all headache on ≥15 days/month and pMOH. We adjusted prevalences for gender and age according to the general population distribution (within the range 18–65 years) in Nepal [20].

We categorized age in five groups (18–25, 26–35, 36–45, 46–55 and 56–65 years). We classed habitation as rural or urban, and categorized altitude of the household as <500 m, 500–999 m, 1,000–1,499 m, 1,500–1,999 m, 2,000-2,499 m or ≥2,500 m. We took household consumption per year in USD (at the time of the survey, USD 1 = NPR 100) as an indicator of the economic wellbeing of the participant and categorized it in three groups: poorest (<USD 950/year); poor (USD 950–1,200/year); intermediate and above (>USD 1,200/year).

We used bivariate and multivariate logistic regression analyses (with odds ratios [ORs] and adjusted ORs [AORs] respectively, each with 95 % CIs) to investigate associations of demographic, lifestyle, environmental and other health factors with each of migraine, TTH and pMOH. Gender, age, household consumption, habitation, altitude, systolic and diastolic BP and EPQRS-N and HADS scores were entered as covariates in the multivariate logistic regression. BP readings and EPQRS-N and HADS scores were treated as continuous variables.

Statistical analyses were carried out using the Statistical Package for Social Science software (IBM SPSS Statistics 21, Chicago, USA).

## Results

The survey was completed by 2,100 participants (1,239 [59.0 %] female, 861 [41.0 %] male) aged 18–65 years (mean age 36.4 ± 12.8 years). There were only nine refusals: hence the participation rate was 99.6 %. Almost two-fifths (822; 39.1 %) were living in households with the poorest economic wellbeing; nearly two thirds (1,328; 63.2 %) were from rural areas; over half (1,100; 52.4 %) were resident above 1,000 m and almost one quarter (470; 22.4 %) lived at or above 2,000 m. The sociodemographic characteristics of the sample are compared with those of the national population (as far as they are available) from the 2011 population and housing census [20] in Table 1.

**Table 1:** Sociodemographic characteristics of the participating sample (*N* = 2,100) and national population

Variable	Sample	National population [20]
	*n* (%)	%
Gender		
Male	861 (41.0)	46.6
Female	1,239 (59.0)	53.4
Age (years)		
18–25	489 (23.3)	27.7
26–35	657 (31.3)	26.1
36–45	438 (20.8)	20.4
46–55	298 (14.2)	14.8
56-65	218 (10.4)	11.0
Household consumption (USD/year)		
<950	822 (39.1)	Data not available
950–1200	806 (38.4)	
>1,200	472 (22.5)	
Habitation		
Rural	1,328 (63.2)	72.8
Urban	772 (36.8)	27.2
Household altitude		
<1,000 m	1,000 (47.6)	Data not available
≥1,000 m	1,100 (52.4)	

### Headache prevalences

#### All headache

Of the 2,100 participants, 1,794 reported headache in the last year. The crude 1-year prevalence of all headache was 85.4 % (95 % CI: 83.9-86.9 %), higher in females (89.2 %) than in males (80.0 %; *p* < 0.001). The gender- and age-adjusted 1-year prevalence was 84.9 %. The age-adjusted female-to-male ratio was 1.1.

#### Migraine

**Fig. 1:**
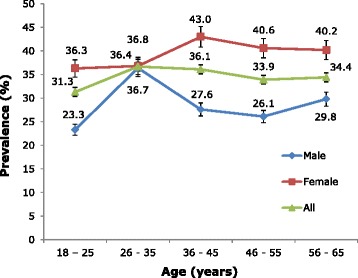
1-year prevalence of migraine by age and gender

The crude 1-year prevalence of migraine was 34.7 % (17.5 % definite, 17.2 % probable). Prevalence was age-related, increasing from young adulthood (18–25 years) in both genders and peaking during 26–35 years among males and 36–45 years among females (Fig. 1).

There was a clear female preponderance (overall 38.2 % versus 28.9 % in males) (Table 2), which is demonstrated in Fig. 1 in all age groups except 26–35 years and was confirmed in both bivariate (OR = 1.6 [95 % CI: 1.3–1.9]; p <0.001) (Table 3) and multivariate regression analysis (AOR = 1.5 [95 % CI: 1.2-1.8]; *p* < 0.001) (Table 4). The gender- and age-adjusted 1-year prevalence was 34.1 % and the age-adjusted female-to-male ratio was 1.3.

**Table 2:** Observed 1-year prevalence (% [95 % CI]) of all headache and headache types by gender, age, household consumption, habitation and altitude

	All headache	Migraine	Tension-type headache	All headache on ≥15 days/month	Probable medication-overuse headache
Gender					
Male	80.0 [77.2–82.6]	28.9 [25.9–31.9]	44.6 [41.3–47.9]	5.1 [3.6–6.6]	1.3 [0.5–2.1]
Female	89.2 [87.3–90.8]	38.2 [35.5–40.9]	38.7 [36.0–41.4]	9.4 [7.8–11.0]	2.8 [1.9–3.7]
Age (years)					
18–25	87.1 [83.7–89.9]	31.3 [27.2–35.4]	48.3 [43.9–52.7]	5.9 [3.8–8.0]	1.0 [0.1–1.9]
26–35	88.4 [85.8–90.7]	36.7 [33.0–40.4]	40.6 [36.8–44.4]	7.8 [5.8–9.8]	2.7 [1.5–3.9]
36–45	81.7 [77.7–85.2]	36.1 [31.6–40.6]	36.8 [32.3–41.3]	7.8 [5.3–10.3]	1.6 [0.4–2.8]
46–55	86.6 [82.1–90.1]	33.9 [28.5–39.3]	41.9 [36.3–47.5]	9.7 [6.3–13.1]	2.6 [0.8–4.4]
56–65	78.4 [72.3–83.6]	34.4 [28.1–40.7]	33.9 [27.6–40.2]	8.3 [4.6–12.0]	3.7 [1.2–6.2]
Household consumption (USD/year)					
950–1,200	85.2 [82.6–87.6]	34.4 [31.1–37.7]	41.4 [38.0–44.8]	7.3 [5.5–9.1]	2.2 [1.2–3.2]
<950	83.5 [80.7–85.9]	35.2 [31.9–38.5]	39.2 [35.9–42.2]	7.2 [5.4–9.0]	1.5 [0.7–2.3]
>1,200	89.2 [85.9–91.7]	34.3 [30.0–38.6]	43.9 [39.4–48.4]	9.1 [6.5–11.7]	3.4 [1.8–5.0]
Habitation					
Rural	83.4 [81.3–85.4]	35.2 [32.6–37.7]	38.0 [35.4–40.6]	8.1 [6.6–9.6]	2.0 [1.3–2.7]
Urban	88.9 [86.4–90.9]	33.8 [30.5–37.1]	46.5 [43.0–50.0]	7.0 [5.1–8.8]	2.6 [1.5–3.7]
Household altitude					
<1,000 m	84.5 [82.3–86.7]	28.7 [25.9–31.5]	46.4 [43.3–49.5]	8.2 [6.5–9.9]	1.8 [1.0–2.6]
≥1,000 m	86.3 [84.3–88.3]	40.1 [37.2–43.0]	36.3 [33.5–39.1]	9.7 [7.9–11.4]	2.5 [1.6–3.4]

**Table 3:** Bivariate logistic regression analyses of associations of each headache type with gender, age, household consumption, habitation and altitude

	Migraine		Tension-type headache		Probable medication-overuse headache	
	OR [95 % CI]	*p*	OR [95 % CI]	*p*	OR [95 % CI]	*p*
Gender						
Male	Reference	–	Reference	–	Reference	–
Female	1.6 [1.3–1.9]	<0.001	0.7 [0.6–0.9]	0.002	2.2 [1.1–4.4]	0.020
Age (years)						
18–25	Reference	–	Reference	–	Reference	–
26–35	1.3 [0.99–1.6]	0.057	0.7 [0.6–0.9]	0.010	2.7 [1.01–7.4]	0.049
36–45	1.2 [0.9–1.6]	0.12	0.6 [0.5–0.8]	<0.001	1.6 [0.5–4.9]	0.44
46–55	1.1 [0.8–1.5]	0.45	0.8 [0.6–1.1]	0.085	2.7 [0.9–8.2]	0.088
56–65	1.1 [0.8–1.6]	0.41	0.6 [0.4–0.8]	<0.001	3.7 [1.2–11.4]	0.023
Household consumption (USD/year)						
950–1,200	Reference	–	Reference	–	Reference	–
<950	1.1 [0.8–1.3]	0.74	0.9 [0.7–1.1]	0.35	0.6 [0.3–1.4]	0.25
>1,200	0.99 [0.8–1.3]	0.99	1.1 [0.9–1.4]	0.40	1.5 [0.8–3.0]	0.22
Habitation						
Rural	Reference	–	Reference	–	Reference	–
Urban	0.9 [0.8–1.1]	0.53	1.4 [1.2–1.7]	<0.001	1.3 [0.7–2.4]	0.34
Household altitude						
<1,000 m	Reference	–	Reference	–	Reference	–
≥1,000 m	1.7 [1.4–2.0]	<0.001	0.7 [0.6–0.8]	<0.001	1.4 [0.8–2.6]	0.25

*OR* odds ratio, *CI* confidence interval

**Table 4:** Multivariate logistic regression analyses of associations of each headache type^a^

	Migraine		Tension–type headache		Probable medication–overuse headache	
	AOR [95 % CI]	*p*	AOR [95 % CI]	*p*	AOR [95 % CI]	*p*
Gender						
Male	Reference	–	Reference	–	Reference	–
Female	1.5 [1.2–1.8]	<0.001	0.7 [0.6–0.9]	0.003	2.6 [1.2–5.3]	0.010
Age (years)						
18–25	Reference	–	Reference	–	Reference	–
26–35	1.3 [1.0–1.6]	0.060	0.8 [0.6–0.9]	0.023	2.7 [0.9–7.5]	0.054
36–45	1.2 [0.9–1.6]	0.19	0.7 [0.5–0.8]	0.004	1.5 [0.5–4.8]	0.52
46–55	1.1 [0.8–1.4]	0.75	0.9 [0.7–1.2]	0.44	2.1 [0.7–6.8]	0.21
56–65	1.1 [0.8–1.6]	0.57	0.6 [0.4–0.9]	0.008	2.6 [0.8–8.8]	0.11
Household consumption (USD/year)						
950–1,200	Reference	–	Reference	–	Reference	–
<950	1.0 [0.8–1.2]	0.94	1.0 [0.8–1.2]	0.76	0.6 [0.3–1.2]	0.16
>1,200	1.1 [0.8–1.4]	0.66	1.1 [0.8–1.2]	0.58	1.3 [0.7–2.4]	0.44
Habitation						
Rural	Reference	–	Reference	–	Reference	–
Urban	1.0 [0.8–1.2]	0.81	1.3 [1.1–1.6]	0.003	1.3 [0.7–2.4]	0.44
Household altitude						
<1,000 m	Reference	–	Reference	–	Reference	–
≥1,000 m	1.6 [1.3–2.0]	<0.001	0.7 [0.6–0.8]	<0.001	1.4 [0.8–2.6]	0.29

^a^Adjusted for gender, age, household consumption, household altitude, systolic and diastolic blood pressure, EPQRS–N and HADS scores; *AOR* adjusted odds ratio, *CI* confidence interval

#### Tension-type headache

The crude 1-year prevalence of TTH was 41.1 % (32.5 % definite, 8.6 % probable). Prevalence was lower in females (38.7 %) than in males (44.6 %; OR = 0.7 [95 % CI: 0.6–0.9]; *p* = 0.002) (Tables 2, 3). The gender- and age-adjusted prevalence was 41.5 % and the age-adjusted female-to-male ratio was 0.86. Prevalence was highest in the age range 18–25 years and decreased with age in both genders, being at its lowest at 56–65 years (OR = 0.6 [95 % CI: 0.4–0.8]; *p* < 0.001) (Table 3). Multivariate regression analysis (Table 4) confirmed that TTH was negatively associated with female gender (AOR = 0.7 [95 % CI: 0.6–0.9]; *p* = 0.003) and age (for age 56–65 years, AOR = 0.6 [95 % CI: 0.4–0.9]; *p* = 0.008).

#### Headache on ≥15 days/month and pMOH

The crude prevalence of all headache on ≥15 days/month was 7.7 % (95 % CI: 6.6-8.8 %), higher in females (9.4 %) than males (5.1 %; *p* < 0.001). The age-adjusted female-to-male ratio was 1.86. The gender- and age-adjusted prevalence was 7.4 %.

Over one quarter (46/161; 28.6 %) of the participants reporting headache on ≥15 days/month fulfilled our criteria for pMOH. The crude prevalence of pMOH was therefore 2.2 % (95 % CI: 1.6-2.8). This disorder was much more common in females (2.8 %) than males (1.3 %; OR = 2.2 [95 % CI: 1.1–4.4]; *p* = 0.020; AOR = 2.6 [95 % CI: 1.2–5.3]; *p* = 0.010). The age-adjusted female-to-male ratio was 2.42 (Tables 2, 3, 4). Prevalence increased with age and was highest in the age range 56–65 years (OR = 3.7 [95 % CI: 1.2–11.4]; *p* = 0.023) (Table 3). The gender- and age-adjusted prevalence was 2.1 %.

The most commonly overused acute treatment was paracetamol in monotherapy (26 cases [56.5 %]). In 6 cases (16.1 %), there was overuse of herbal medicines, usually navaratna sancho and zandubalm, which are customarily inhaled or administered via the nasal mucosa for treatment of headache in Nepal.

### Associations with household consumption, habitation, and household altitude

None of the headache disorders was associated with household consumption (Table 4). TTH was weakly associated with urban dwelling (OR = 1.4 [95 % CI: 1.2–1.7]; *p* = 0.001; AOR = 1.3 [95 % CI: 1.1-1.6]; *p* = 0.003) (Tables 3 and 4). Migraine was strongly associated with living at an altitude of ≥1,000 m in both bivariate (OR = 1.7 [95 % CI: 1.4-2.0]; *p* < 0.001) (Table 3) and multivariate analyses (AOR = 1.6 [95 % CI: 1.3–2.0]; *p* < 0.001) (Table 4). In view of this finding, we estimated the prevalence of migraine by altitude category, standardising for age and gender against census data for the Nepali population [20] (Table 5). This analysis revealed that the age- and gender standardised prevalence of migraine was 27.9 % in the low-lying Terai (<500 m; there were no participants in our sample living between 500 and 999 m). Prevalence increased in an almost linear relationship with altitude up to 2,000 m. Thereafter it levelled, and indeed declined. This relationship was evident in both genders.

**Table 5:** Observed and age- and gender-standardised prevalence of migraine by altitude and gender

Physiographic division and altitude	Observed prevalence in sample				Standardised prevalence* [20]		
	*N*	All *n* (%)	Male *n* (%)	Female *n* (%)	All %	Male %	Female %
Terai <500 m	1,000	287 (28.7)	86 (22.0)	201 (33.0)	27.9	22.1	32.9
Hill							
1,000–1,499 m	470	176 (37.7)	57 (31.1)	119 (41.5)	36.5	31.6	40.7
1,500–1,999 m	160	68 (42.5)	31 (39.7)	37 (45.1)	44.4	41.3	47.0
Mountain							
2,000–2,499 m	254	116 (45.7)	46 (41.1)	70 (49.3)	45.5	40.8	49.6
≥2,500 m	216	81 (37.5)	29 (29.9)	52 (43.7)	37.9	31.4	43.6

*Age- and gender-standardised against census data for the Nepali population [20]

A negative association between TTH and altitude was indicated by both bivariate (OR = 0.7 [95 % CI: 0.6–0.8]; *p* < 0.001) and multivariate analyses (AOR = 0.7 [95 % CI: 0.6–0.8]; p < 0.001) (Tables 3 and 4).

## Discussion

We found a gender- and age adjusted 1-year prevalence of all headache of 84.9 %, of migraine 34.1 %, of TTH 41.5 %, of all headache on ≥15 days/month 7.4 %, and of pMOH 2.1 %. We showed that living at an altitude of ≥1,000 m was highly associated with migraine, and urban dwelling was less strongly associated with TTH.

Before discussing the individual headache types, we note that our study used tried and tested methods [21]. We randomly selected from the whole of Nepal, while the very high participation rate (>99 %) achieved through careful methodology [10, 15] effectively excluded participation bias. Face-to-face interviews were conducted carefully to ensure there were no missing data. These were considerable strengths of the study. There were, however, some important limitations, which we will draw attention to in the context of their relevance.

### Prevalence of migraine

Our most obvious finding was that the prevalence of migraine in Nepal is very much higher than the mean global estimate of 14.7 % [22]. Our first comment on this is to note that more recent studies, in all regions except the Far East, have generally yielded higher values than 14.7 %. The mean global estimate is based on a large number of heterogeneous studies, performed with varying methods during a period of >30 years. Many reports are silent on the crucial issue of how they applied diagnostic criteria with respect to definite and probable migraine; while some explicitly excluded the latter, it is highly probable that more did so without making this evident, because only recently has there been clear guidance and explanation of why this is misleading [21]. The consequences, in our view, are that many of these studies significantly underestimate migraine prevalence and, therefore, so does the global mean. Nonetheless, our finding of 34.1 % in Nepal is considerably higher even than the 25.2 % reported from neighbouring India [23] (although this was from the single State of Karnataka in the south). We used the same methodology and diagnostic questionnaire as Karnataka [24]; indeed, LTB has supported studies using similar methods and the same questionnaire in many other countries, cultures and languages [13]: in Russia [25], China [26], Zambia [27], Ethiopia (unpublished), Pakistan (unpublished), Saudi Arabia (unpublished), Morocco (unpublished). None has discovered such a high prevalence of migraine; in fact, India (Karnataka) was the highest [23].

Nepal is a poor country, but we discovered no association with economic wellbeing that might offer an explanation (neither was there one in India [23]). The clearly relevant factor is altitude: over half our sample were resident at or above 1,000 m, and nearly one quarter above 2,000 m. Altitude, we discovered, is an environmental factor so strongly associated with migraine that living above 1,000 m led to 60 % increased odds of having the disorder. In practice this meant that, among the adult population living above this altitude, an additional 11.4 % had migraine (Table 2). Since more than half the population were in fact living above 1,000 m, this explained a very large part of the overall excess prevalence. For the remainder, we should look to the distinctive age and gender distributions of Nepal: more precisely, to the high proportions of young adults (median age is 22 years [9]), and females among these adults, the two strongest determinants of migraine prevalence [1]. Furthermore, large numbers of healthy people have consistently been going abroad for work, around half of them in the age group 15–35 years, and mostly males [9], leaving behind a skewed adult distribution perhaps more at risk of migraine. It is entirely possible to see this as the explanation of the difference between 27.9 %, the standardised prevalence in the Terai of Nepal, and 25.2 %, the standardised prevalence in Karnataka [23] (which is at a mean altitude of about 900 m [28]).

We should acknowledge two diagnostic issues. First, it was a limitation of the study that we were not able to perform a prior validation of the headache diagnostic questionnaire in its Nepali translation. Because there are no headache specialists in the country, we had no means of applying a “gold standard” for this purpose [21]. We had to rely on the fact that the questionnaire had been validated in many other languages and countries [13], including India with not too dissimilar cultural settings [24].

Second, having said this, we encountered a problem that was not met in India: such a large proportion (75.8 %) of respondents with any type of headache reported photophobia that this symptom had virtually no discriminative value as a diagnostic criterion, and we could not use it within the framework of ICHD [18]. Photophobia is a technical concept, not easy to convey to lay participants (even by trained interviewers) [21]. Because of this we had taken great care in translation to convey not merely an aversion to bright light but the idea of a wish to withdraw into darkness from ordinary light. Our eventual solution was to disregard photophobia altogether, and in our view this was necessary: the prevalence estimate for migraine would otherwise have been much higher. What this suggests is that ICHD criteria – or at least this particular one – may not be universally applicable, and not for linguistic reasons.

### Other headache types

The estimated prevalence of TTH in Nepal at 41.1 % is in line with the estimated global average [2], although considerably lower than some national estimates [29, 30]. Two factors are relevant here. One is whether or not survey participants report infrequent TTH, which can have a marked effect on the prevalence estimate and is likely to be determined in part culturally and in part by the insistence (and purpose) of the interviewer [23, 24]. In our case, not only did we not consider infrequent TTH to be of public-health significance, but also we focused only on the most bothersome headache in participants identifying more than one type. Those with both migraine and TTH would tend to regard the former as the more bothersome [31], leading to a partial neglect of TTH. Our prevalence estimate for TTH was therefore somewhat conservative.

We suspect this was a factor in the observed negative association between TTH prevalence and altitude: as migraine prevalence increased with altitude, reporting of TTH became less likely. In other words, it was artefactual. We do not know this, but on the other hand can offer no explanation for a true negative association.

TTH was significantly more prevalent in urban areas, an association not observed for migraine or pMOH. Possible explanations for the higher prevalence of TTH may be the noisy and stressful environments of the cities. Also, physical inactivity is a risk factor specifically for non-migraine headache [32], and people in rural areas in Nepal are more physically active working in the fields. These are speculative proposals.

According to our estimate, the prevalence of headache on ≥15 days/month in Nepal is more than twice the global average [2] and much higher than in neighbouring China [26] and India [23]. Several factors such as low socio-economic status, poor access to health services with a paucity of health-care providers and lack of standard protocols for diagnosis and treatment of headache disorders, all against a background of high levels of episodic headache, offer what may be sufficient explanation.

We do not know what the cases with headache on ≥15 days/month were diagnostically, other than the proportion diagnosed as pMOH. We estimated the prevalence of pMOH in Nepal at 2.1 %, which is towards the upper end of the range for most countries studied (0.5–2.6 %) [33], and much higher than the 0.6 % and 1.2 % in neighbouring China [26] and India [23]. Surprisingly, the proportion of pMOH among those with headache on ≥15 days/month was only 29 % (46/161); in other countries where studies have used similar methodology, this proportion is closer to two thirds [23, 25, 26]. This may be due to poverty: most pMOH was associated with overuse of simple analgesics sold over-the-counter (OTC), but many people in Nepal cannot afford these. They have recourse to alternative treatments, which may include plant-based remedies that are used regularly across the country, especially in the rural areas. Many of these have phytochemical or pharmacological properties [34], and may be able to cause transformation of episodic headache to pMOH. We endeavoured to include these in our enquiry, and identified six cases, but herb-based remedies may not be perceived by lay people as therapy for headache and it is likely that they were underreported.

### Implications for Nepal

Headache disorders are very common in Nepal. Only the prevalence of TTH is in line with the global average. That of headache on ≥15 days/month is double, while the prevalence of pMOH is towards the upper end of the range for most countries studied. The prevalence of migraine, however, appears uniquely high, explained, we believe, by some of the distinct characteristics of this country. One is the combination of mountainous and hilly terrain over much of the country, important in the light of the previously unreported but strong association between migraine prevalence and altitude. Another is the demographic make-up.

The new evidence from this study will inform national health policy, and provide a basis for health-care needs assessment. Meanwhile, research of a different type is needed to find explanations for the association between migraine and altitude, since these may be relevant to health-care interventions. Continuous long-duration exposure to high altitude compromises oxygen uptake and results in haemodynamic changes, with elevated haemoglobin levels, increased blood viscosity and reduced oxygen delivery to brain tissues [35, 36]. Migraine has been linked with the consequences of such changes [37–40], but their relevance at lower altitudes, between 500 and 2,000 m, needs to be investigated.

## Conclusion

Headache disorders are very common in Nepal. Migraine is unusually so, and strongly associated with altitude of dwelling, which in this mountainous country largely explains the high national prevalence. Headache occurring on ≥15 days/month is also very common in comparison with the world average. This new evidence will inform national health policy and provide a basis for health-care needs assessment. However, research is needed to explain the association of migraine with altitude, since it may be relevant to health-care interventions.

## Abbreviations

AOR: Adjusted odds ratio
BMI: Body-mass index
BP: Blood pressure
CI: Confidence interval
EPQRS-N: Eysenck Personality Questionnaire Short Form Revised version- Neuroticism
GBD: Global burden of disease
HADS: Hospital Anxiety and Depression Scale
HARDSHIP: Headache-Attributed Restriction, Disability, Social Handicap and Impaired Participation
ICHD: International classification of headache disorders
IRC-KUSMS: The Institutional Review Committee of Kathmandu University School of Medical Sciences, Dhulikhel Hospital
LTB: *Lifting The Burden*
MOH: Medication-overuse headache
NPR: Nepalese rupee
OR: Odds ratio
OTC: Over-the-counter
pMOH: Probable MOH
SEAR: South-East Asia Region
SPSS: Statistical package for social science
TTH: Tension-type headache
USD: United States dollar
YLD: Year of life lost to disability
